# Time-dependent viral interference between influenza virus and coronavirus in the infection of differentiated porcine airway epithelial cells

**DOI:** 10.1080/21505594.2021.1911148

**Published:** 2021-05-25

**Authors:** Ju-Yi Peng, Dai-Lun Shin, Guangxing Li, Nai-Huei Wu, Georg Herrler

**Affiliations:** a Institute of Virology, University of Veterinary Medicine Hannover, Hannover, Germany; b College of Veterinary Medicine, Northeast Agricultural University, Harbin, China; c Department of Veterinary Medicine, National Taiwan University, Taipei, Taiwan

**Keywords:** Co-infection, super-infection, viral interference, porcine respiratory coronavirus, swine influenza virus, air-liquid interface culture, porcine respiratory disease complex, porcine aminopeptidase N, innate immune response, receptor

## Abstract

Coronaviruses and influenza viruses are circulating in humans and animals all over the world. Co-infection with these two viruses may aggravate clinical signs. However, the molecular mechanisms of co-infections by these two viruses are incompletely understood. In this study, we applied air-liquid interface (ALI) cultures of well-differentiated porcine tracheal epithelial cells (PTECs) to analyze the co-infection by a swine influenza virus (SIV, H3N2 subtype) and porcine respiratory coronavirus (PRCoV) at different time intervals. Our results revealed that in short-term intervals, prior infection by influenza virus caused complete inhibition of coronavirus infection, while in long-term intervals, some coronavirus replication was detectable. The influenza virus infection resulted in (i) an upregulation of porcine aminopeptidase N, the cellular receptor for PRCoV and (ii) in the induction of an innate immune response which was responsible for the inhibition of PRCoV replication. By contrast, prior infection by coronavirus only caused a slight inhibition of influenza virus replication. Taken together, the timing and the order of virus infection are important determinants in co-infections. This study is the first to show the impact of SIV and PRCoV co- and super-infection on the cellular level. Our results have implications also for human viruses, including potential co-infections by SARS-CoV-2 and seasonal influenza viruses.

## Introduction

Coronaviruses and influenza viruses are major respiratory pathogens co-circulating in animals and humans [1,2]. Porcine respiratory coronavirus (PRCoV) and swine influenza viruses (SIV) are circulating in pig populations, rendering co-infections likely to occur [3]. The pathogenesis of coronavirus and influenza virus infections in pigs resembles those of these two pathogens in humans in many aspects [1,4]. PRCoV shares some characteristics with SARS-CoV including the bronchointerstitial pneumonia, long-periods replication in the lung, and a two-phase inflammatory process [5,6]. Influenza virus infection in swine has many similarities with that in humans including the same antigenic subtypes, repeated genetic exchange of viruses among two host species, and the clinical manifestation [4,7,8]. Therefore, the infection of PRCoV and SIV in porcine models has been used to mimic the infection of coronavirus and influenza virus in humans [4,7].

With the high prevalence of influenza viruses and coronaviruses, it is very common to detect two viruses together [3,9]. However, a substantial amount of research only focused on epidemiology and case reports [3,10,11]. Very little is known about how co- and super-infections by SIV and PRCoV affect pathogenesis [12]. Experimentally, viral interference has been shown to occur during co- or superinfections by PRCoV and SIV in pigs [13]. In nasal swabs, the amount of SIV was decreased and no PRCoV was detected in the co-infection group as compared to the mono- infection group [13,14]. Furthermore, in co-infections of porcine precision-cut lung slices (PCLS) a lower amount of PRCoV was detected than after mono-infection [15]. Taken together, the previous studies indicated an interference between the two viruses. Nevertheless, the mechanisms of the viral interference between SIV and PRCoV are still unknown. To further study this phenomenon, we applied an *in vitro* model of primary cultures of porcine tracheal epithelial cells (PTECs) grown under air-liquid interface (ALI) conditions. Compared with conventional primary cells grown on plastic, PTECs maintained in ALI conditions differentiate into specialized epithelial cells, including ciliated and mucus-producing cells [16,17]. Furthermore, PTECs grown in ALI cultures have a long life span and regenerative characteristics [18]. Until now, PTECs have been analyzed in mono-infections by respiratory viruses and co-infections with bacteria [19,20].

Recently, we compared the virulence of SIV and PRCoV, respectively, in ALI culture systems and highlighted virus-specific infection signatures [16,21]. In contrast to the infection by PRCoV, infection of ALI cultures by SIV induced the loss of ciliated cells due to apoptosis [16]. In the course of a seven day period of infection, the number of ciliated cells decreases without affecting the barrier function as indicated by the transepithelial electrical resistance. This finding indicates that in the infected area, the loss of ciliated cells is compensated by other cells, presumably by basal cells that have started to differentiate into specialized cells. In the transition time until they are well-differentiated, i.e. ciliated, they were shown to have different surface properties [16]. These observations are indicative of a regeneration process that is occurring to repair the virus-induced damage. Changes on the cell surface during the regeneration phase may also affect the susceptibility to virus infection. Therefore, we were interested to know whether prior infection by SIV will affect the secondary infection by PRCoV.

In the present research, we aimed to investigate the interactions between SIV and PRCoV in the context of co- and super-infections of PTECs by the two viruses within different interval days. Co-infection means that PTECs are simultaneously infected by SIV and PRCoV, and super-infection means that the primary virus infects PTECs prior to the secondary virus. We also addressed the mechanisms of interference. Our findings suggest a scenario of viral-viral interactions in the coinfection of the airway that may also be relevant for humans.

## Materials and methods

- Primary porcine tracheal epithelial cells (PTECs)

The tracheas were collected from pigs obtained from a local slaughterhouse. PTECs were harvested from the porcine trachea as previously described [16,21]. Briefly, PTECs were initially maintained in bronchial epithelial cell growth medium (Lonza). After PTECs had reached 80% confluence, PTECs were transferred to transwell filters and maintained with ALI medium under air-liquid interface conditions for at least 3 weeks. All cells were tested negative for porcine circovirus-2, porcine reproductive and respiratory syndrome virus, porcine cytomegalovirus, porcine influenza A virus, porcine respiratory coronavirus, *Mycoplasma hyorhinis*, and *Mycoplasma hyopneumoniae* by multiplex Polymerase Chain Reaction. The results were repeated at least with six PTECs from three independent donors, three fields per culture.

- Cells and viruses

Swine testicular (ST) cells and Madin–Darby canine kidney cells (MDCK) were maintained in Eagle’s minimal essential medium (EMEM; PAN-Biotech) supplemented with 10% fetal calf serum (FCS). Both cell types were incubated in a humidified atmosphere containing 5% CO_2_ at 37°C and passaged every 2 to 3 days. PRCoV Bel85 was obtained from Prof. Luis Enjuanes, Campus Universidad Autónoma de Madrid, Spain. Swine influenza virus of subtype H3N2 (A/sw/Herford/IDT5932/2007, H3N2) was provided by Prof. Michaela Schmidtke, University of Jena, Germany. All stocks were propagated on ST cells (PRCoV) or MDCK cells (influenza virus), respectively, in EMEM. After incubation for 36 to 48 hr at 37°C, the supernatants were harvested and stored at −80°C.

- Virus infection of differentiated epithelial cells

The viral inoculation of PTECs was performed as previously described [16]. Briefly, PTECs were apically inoculated with PRCoV or SIV at 1 × 10^3^ focus forming units (FFU) and 4 × 10^4^ FFU, respectively. After 2 h of incubation at 37 °C, PTECs were washed with PBS twice to remove unbound viral particles and fresh ALI medium was added. At the time of measurement, apically released viral particles were collected by overlaying the cells with medium for 30 min for virus harvesting. The virus replication kinetics were determined by performing a focus-forming assay.

- Immunofluorescence analysis (IFA)

For analyzing the susceptibility of ciliated cells to PRCoV, the samples were fixed with 3% paraformaldehyde (PFA) for 1 hr and permeabilized with 0.5% Triton X-100 for 20 min at room temperature. The samples were blocked with 1% BSA, then incubated with a monoclonal mouse anti-coronavirus-antibody (FIPV3-70; 1:1000, Thermo Fischer, Scientific) followed by Alexa Fluor® 488 conjugated secondary antibody staining (Thermo Fisher). The primary antibody is directed to the nucleocapsid protein of feline coronavirus and related porcine coronaviruses [15,21]. Ciliated cells were stained by Cy3-labeled antibody against β-tubulin (1:400; Sigma-Aldrich, MO, USA).

For analyzing the expression of porcine aminopeptidase N (pAPN), the samples were fixed with 3% PFA and then blocked with 1%BSA. Subsequently, the samples are stained by rabbit anti-pAPN antibody [22] followed by Alexa Fluor® 488 conjugated secondary antibody staining (Thermo Fisher). In the next steps, the samples were fixed with 3% PFA again and permeabilized with 0.2% Triton X-100 for 7 min at room temperature. Ciliated cells were stained by Cy3-labeled antibody against β-tubulin (Sigma-Aldrich, St. Louis, MO, USA). The nuclei were stained by 1 μg/ml DAPI (4′,6-diamidino-2-phenylindole), and the membrane of the transwell filters was embedded with ProLong® Gold Mountant (Life Technologies). Confocal immunofluorescence microscopy of samples was performed using a TCS SP5 confocal laser scanning microscope equipped with a 63× (NA, 1.40) oil HCX PL Apo objective (Leica).

- Virus titration

To determine the infectivity of the harvested supernatants, a focus-forming assay was performed as described previously with some modifications [23]. Briefly, ST and MDCK cells were seeded in 96-well plates one day before the experiment. Serial 10-fold dilutions of samples harvested from ALI cultures were performed and then inoculated on cells for 1 hr at 37 °C. Cells were overlaid with Avicell. After incubation for 24 h at 37°C, cells were washed, fixed with 3.7% formalin, and permeabilized with quencher buffer (0.5% Triton X-100 with 20 mM glycine in PBS). A primary anti-coronavirus-antibody (FIPV3-70; 1:1000) and anti-influenza antibody (1:1000, AbDSeroTec) was added for I hr at room temperature, followed by a secondary horseradish peroxidase (HRP) antibody (SeraCare KPL) for I hr at room temperature. Subsequently, a substrate (True Blue; KPL) was used for immunological staining. The calculated virus titer is indicated in FFU per ml (FFU/ml). The detection limit was 200 infectious particles. Therefore, the samples below the detection level were set to 100 FFU.

- Quantitative RT-PCR

To analyze the innate immune response of the PTECs, real-time PCR was performed as previously described with a slight modification [19]. Cells were infected apically with 4 × 10^3^ FFU SIV or pre-treatment with poly (I:C). One day later, the membrane was cut down and lysied in RLT lysis buffer (Qiagen). Total RNA was isolated by using RNeasy Minikit® according to the manufacturer’s instructions (Qiagen). Then, real-time PCR reaction was performed using QuantiTect SYBR Green PCR Kit (Qiagen) according to the manufacturer’s instructions. Primers and references for detecting swine Mx1, ISG15, IFNβ, and β-actin transcripts were listed in the previous study [19].

- Statistical analyses

If not stated otherwise, experiments were performed at least four to six times with samples derived from two to three donors. Results are expressed as the means with standard deviations. Data were analyzed by one-way-ANOVA and Tukey multiple comparison test, using GraphPad Prism (version 5) software. A *P* value of <0.05 was considered significant.

## Results

### Prior infection with SIV interferes with the replication of PRCoV

**Figure 1.**
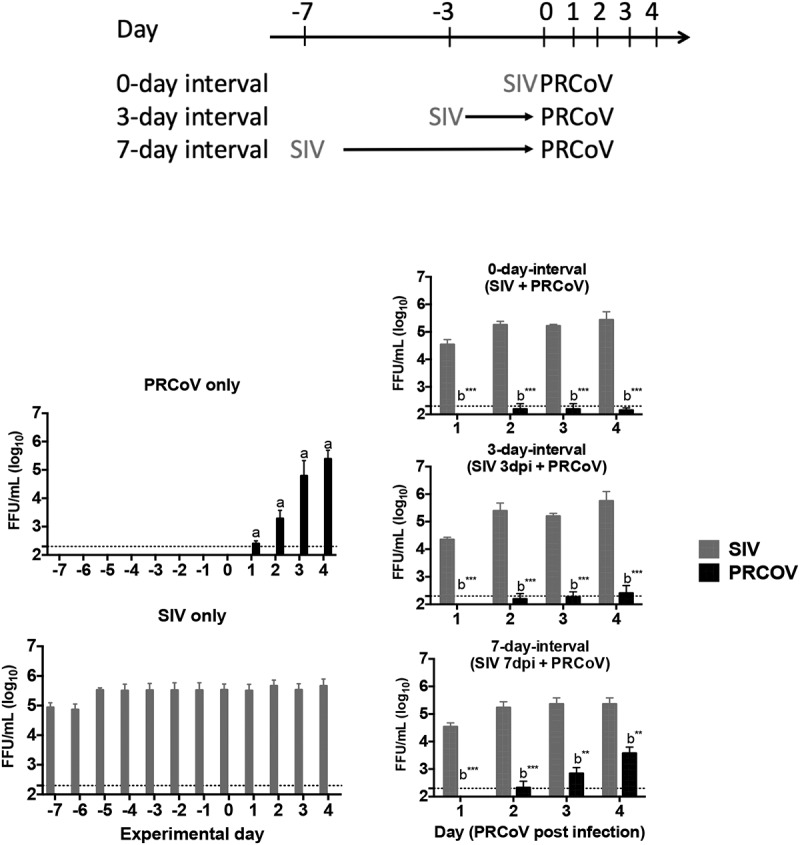
**Virus release from porcine primary tracheal epithelial cells (PTECs) infected by swine influenza virus, followed at intervals of 0, 3, or 7 days by porcine respiratory coronavirus (PRCoV)**. PTECs were first infected with 4 × 10^4^ FFU of SIV followed by infection with 1 × 10^3^ FFU PRCoV 0, 3, or 7 days later. Control PTECs were infected with SIV or PRCoV only. Viruses released from the apical side were harvested at different time points and titrated by focus-forming assay in ST cells and MDCK cells, respectively. The dashed lines indicate detection limits for the assays. The results are shown as means ± SEM and significance. a, b significant differences between groups are indicated with different letters (** *p* < 0.01, *** *p* < 0.001)

In our previous study, infection of PTECs by SIV caused the most severe loss of ciliated cells at 7 dpi. As this effect is of major importance for our study, it is illustrated in Fig. S1. The loss of ciliated cells is compensated by basal cells that start to differentiate into specialized cells, indicative of a regenerative process [16]. During a transition phase, the epithelial cells differ from well-differentiated cells in the expression of APN, the cellular receptor for PRCoV [16]. In addition, the immune response and clinical signs in pigs are decreased at day 3 after SIV infection [24]. Thus, we designed the 0-, 3- and 7-day intervals to analyze the scenario of co- and super-infection by SIV and PRCoV (Figure 1). To investigate the effect of influenza virus infection on secondary virus infection, PTECs were first infected with SIV followed by PRCoV after the respective interval. Viruses released from the apical side were harvested at different time points and titrated by focus-forming assay with ST cells and MDCK cells, respectively. As shown in Figure 1 and Table 1, in the course of a mono-infection, the titer of PRCoV increased progressively from day one to day four reaching a titer of approximately 10^6^ FFU/ml. The maximum titer of SIV in mono-infection, approximately 10^5^ FFU/ml, was reached already at 1 day post infection (dpi) and remained stable until 10 dpi. As for the co- and super-infection, different outcomes were observed with different interval days. In the 0-day-interval group (co-infection), when PTECs were simultaneously infected with SIV and PRCoV, very significant inhibition of PRCoV amplification was determined from 1 to 4 dpi, compared with mono-infection of PRCoV (*p* < 0.001). Similarly, in the 3-day-interval group (super-infection), very significant inhibition of PRCoV growth was seen from 1 dpi to 4 dpi, compared with mono-infection of PRCoV (*p* < 0.001). As for the 7-day-interval (super-infection), some replication of PRCoV was detected at 3 dpi, and a titer of almost 10^4^ FFU/ml was reached at 4 dpi (*p* < 0.01). In these experiments, SIV was applied at a dose of 4 × 10^4^ FFU. This amount of virus selected after a series of initial experiments, where a tenfold higher as well as a tenfold lower dose had been analyzed. A dose of 4 × 10^5^ FFU was excluded from our experimental approach, because it resulted in a detrimental effect on the barrier function as indicated by the loss of the transepithelial electrical resistance. As shown in Fig. S2, a dose of 4 × 10^3^ had a similar effect on the co-infection by PRCoV as a tenfold higher dose.

**Table 1.** The summarized viral titers after secondary pathogen infection

Interval day	Mono/co-infection	SIV titer on each day				PRCoV titer on each day					
		1	2	3	4	1	2		3		4
	Prior infection of SIV										
-	SIV	+++	+++	+++	+++	-	-		-		-
-	PRCoV	-	-	-	-	+	+		++		++++
0	SIV + PRCoV	++	+++	+++	+++	-	-		-		-
3	SIV 3dpi + PRCoV	++	+++	+++	+++	-	-		-		-
7	SIV 7dpi + PRCoV	++	+++	+++	+++	-	-		+		+
	Prior infection of PRCoV										
-	PRCoV	-	-	-	-	+++		+++		+++	+++
-	SIV	+++	+++	+++	+++	-		-		-	-
3	PRCoV 3dpi + SIV	++	++	++	++	++		++		++	++

+++: 10^5^ to 10^6^ FFU/ml, ++:10^3^ to 10^5^ FFU/ml, +:2.3X10^2^ to 10^3^ FFU/ml, -: less than 2.3 × 10^2^ FFU/ml

### The detrimental effects of SIV-PRCoV coinfection on ciliated cells

**Figure 2.**
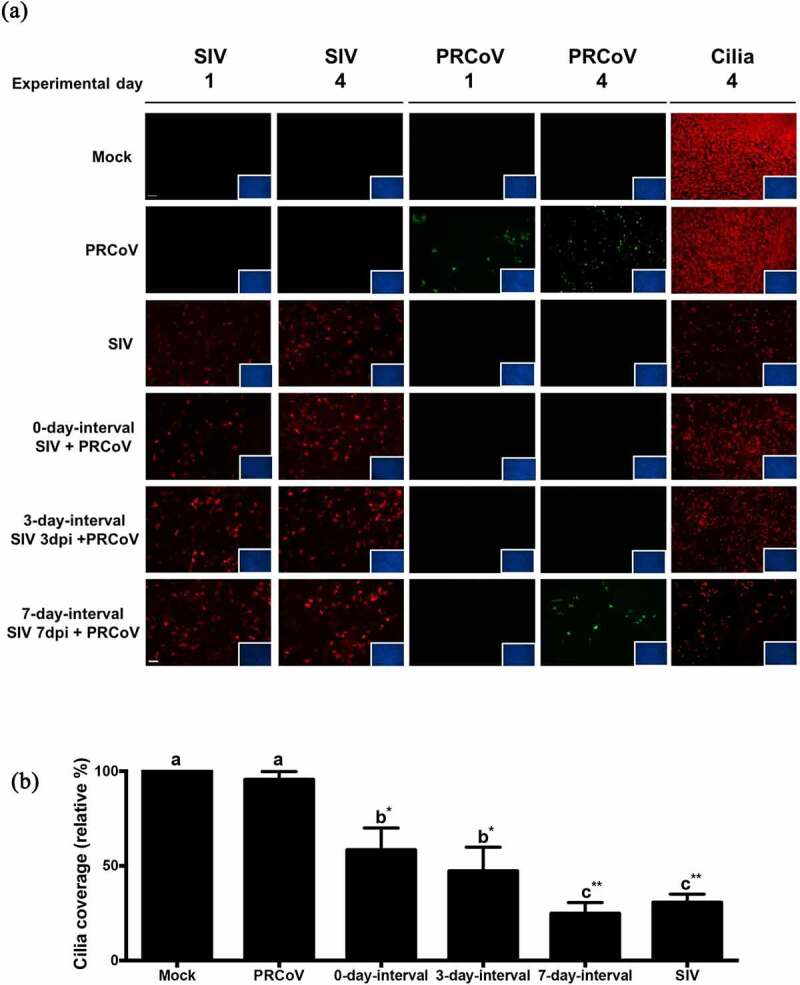
**The correlation of PRCoV susceptibility and ciliated cell effected by the prior infection by SIV**. (a) PTECs were infected by SIV followed at intervals of 0, 3, or 7 days by PRCoV. The cells were fixed at 1 or 4 days post-infection by PRCoV to determine the loss of cilia after SIV infection. Red: influenza nucleoprotein, Green: CoV nucleocapsid, Red: ciliated cells, (b) Detrimental effect on PTECs. The average coverage of ciliated cells was determined by using ImageJ and shown in bar chart; mock-infected group was normalized to 100%. The results are shown as means ± SEM and significance. a, b significant differences between groups are indicated with different letters (** *p* < 0.01, *** *p* < 0.001)

To get information about the detrimental effects of the infection by SIV at different interval times, we determined the loss of ciliated cells by monitoring changes in β-tubulin staining and quantifying the coverage of ciliated cell. As shown in Figure 2a, infection by SIV resulted in a reduction of the cilia coverage. The longer interval time caused a more pronounced loss of ciliated cells. The most dramatic loss of the cilia staining was observed at the 7-day-interval group compared to the samples of the 3-day and 0-day-interval. No PRCoV antigen was detected at the 0-day and 3-day-interval; at the 7-day-interval, a low number of PRCoV-infected cells was observed. DAPI-staining of nuclei indicated that the virus-infected PTECs were still present as a confluent cell layer. To get more exact data about the loss of ciliated cells, the fluorescent signals were quantified by comparing the infected cells with the mock sample (set as 100%). As shown in Figure 2b, the value of remaining ciliated cells at the 7-day-interval is 24.8% (*p* < 0.001), at the 3-day-interval 51.8%(*p* < 0.01), and at the 0-day-interval 58.3% (*p* < 0.01).

### SIV infection of PTEC results in enhanced expression of pAPN

**Figure 3.**
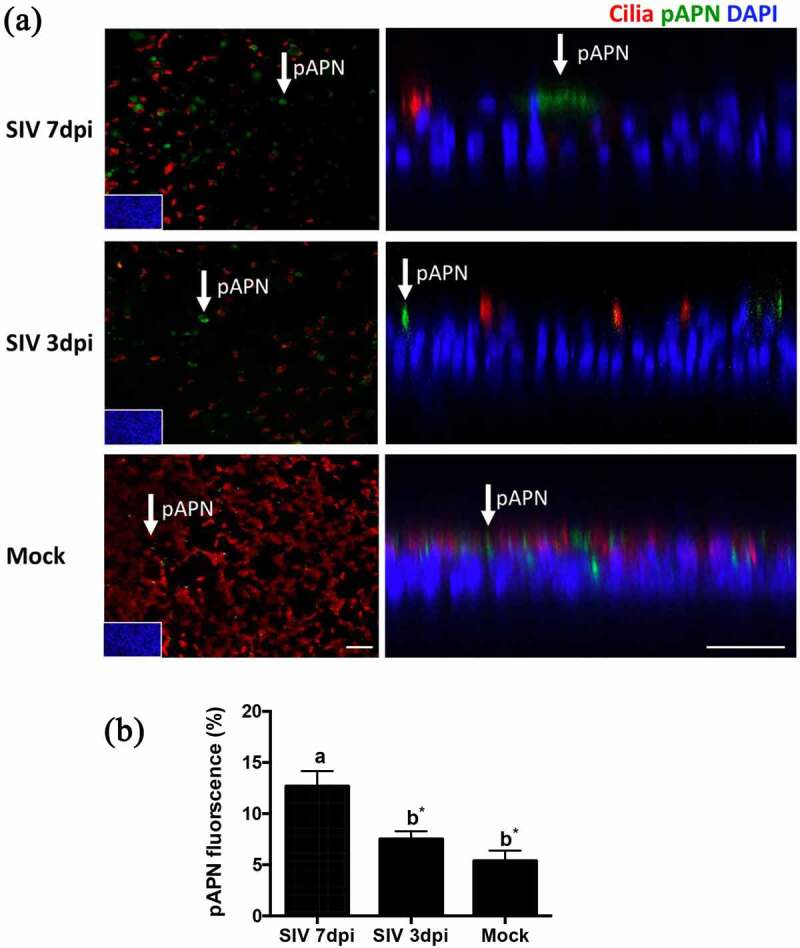
**The expression of pAPN on PTECs after infection by SIV**. (a) PTECs were apically infected with SIV or mock-infected. The cells were fixed at 7 or 3 days post-infection to determine the expression of pAPN by immunofluorescent staining. Red: ciliated cells, Green: pAPN, Blue: nuclei. (b) To quantify the expression of pAPN, the areas containing green fluorescent were determined by ImageJ. Statistical analysis was performed by one-way ANOVA and followed by Tukey’s multiple comparisons test. The results are shown as means ± SEM and significance. a, b significant differences between groups are indicated with different letters (* *p* < 0.05)

A previous study has revealed that the loss of ciliated cells observed after infection by SIV is associated with an enhanced expression of α2,3-linked sialic acids [12]. The different expression of receptors may have relevance for viral-coinfection. To investigate whether SIV infection of PTECs affects the expression of pAPN, we performed an immunofluorescence analysis to visualize pAPN and ciliated cells. PTECs were fixed at 3 dpi or 7 dpi after SIV infection or without SIV infection. Figure 3 shows that SIV infection of PTEC indeed affects the expression of pAPN (green fluorescence). In PTECs without SIV infection, the expression of pAPN is presented as pin-point shaped fluorescent signals that are diffusely distributed. For the PTECs infected by SIV, the expression of pAPN is presented as large spots and mostly located in areas of non-ciliated cells. Quantification of the fluorescent signals on epithelial cells indicated that the expression of pAPN at 7 dpi was higher than at 3 dpi and mock (*p* < 0.05).

### Influenza virus infection enhances the innate immune response

**Figure 4.**
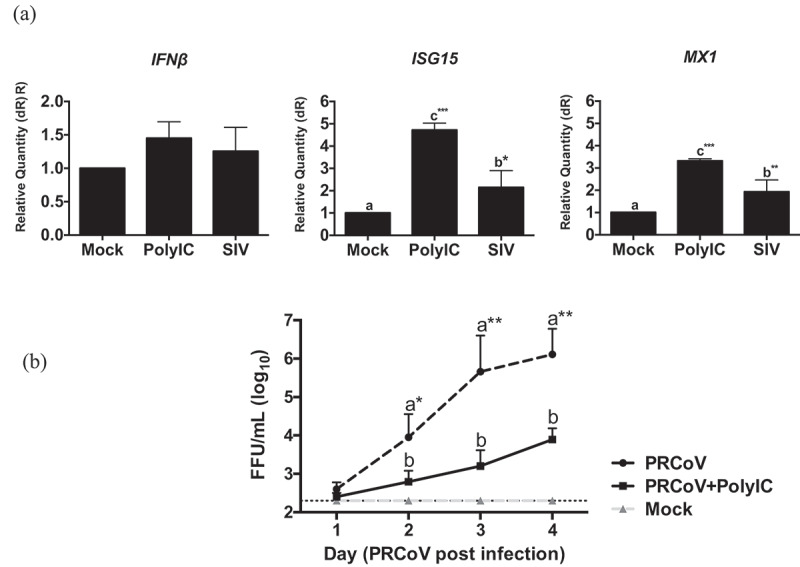
**Innate immune response in PTECs after SIV infection and consequence for PRCoV replication**. (a) The relative quantity of *IFNβ, ISG15*, and *MX1* genes on PTECs after SIV infection or Poly (I:C) pretreatment. PTECs were infected or Poly (I:C) pretreated for 2 h from the apical surface. At 24 h post-infection, the messenger RNA from each group was used to perform quantitative real-time PCR. *IFNβ, Mx1*, and *ISG15* genes were normalized to β-actin expression. The calculation of 2− ΔΔCT method was normalized to mock-infected groups. (b) Effect of Poly (I:C) on PRCoV. With or without one-day Poly (I:C) pretreatment, PTECs were infected by PRCoV. The results were shown as means ± SEM determined. a, b, c significant differences between groups are indicated with different letters (* p < 0.05, ** *p* < 0.01 or *** *p* < 0.001)

Innate immunity has been identified as a protective factor against coronavirus infections [13]. SIV is able to induce innate immunity-related cytokines in PTECs [10]. The relative expression of the interferon-stimulated genes, such as the upregulation of *Mx1* and *ISG15* genes, was used to reflect the host response to influenza virus-infected cells. To understand the innate immune response to SIV on PTECs, PTECs were infected by SIV. At 24 h post-virus infection, the cells were collected from filter supports, and the total RNA was extracted. As shown in Figure 4a, one step quantitative real-time PCR results showed that *IFNβ* was upregulated to some extent. The expression of *ISGs* and *MX1* were significantly increased in SIV-infected cells compared to the mock-infected sample (*p* < 0.05 and *p* < 0.01, respectively).

Next, we were interested to know whether the innate immune response inhibits the replication of PRCoV. We used poly (I:C) to stimulate an innate immune reaction. PTECs were pretreated with poly (I:C) 1 μg/well one day before infection by PRCoV. Compared to PTECs without Poly (I:C) pretreatment, the replication of PRCoV was significantly inhibited by Poly (I:C) (Figure 4b). These results suggest that – similar to the poly (I:C) effect, SIV may inhibit the replication of PRCoV by inducing an innate immune response.

### Prior infection of PRCoV has little effect on the replication of SIV

**Figure 5.**
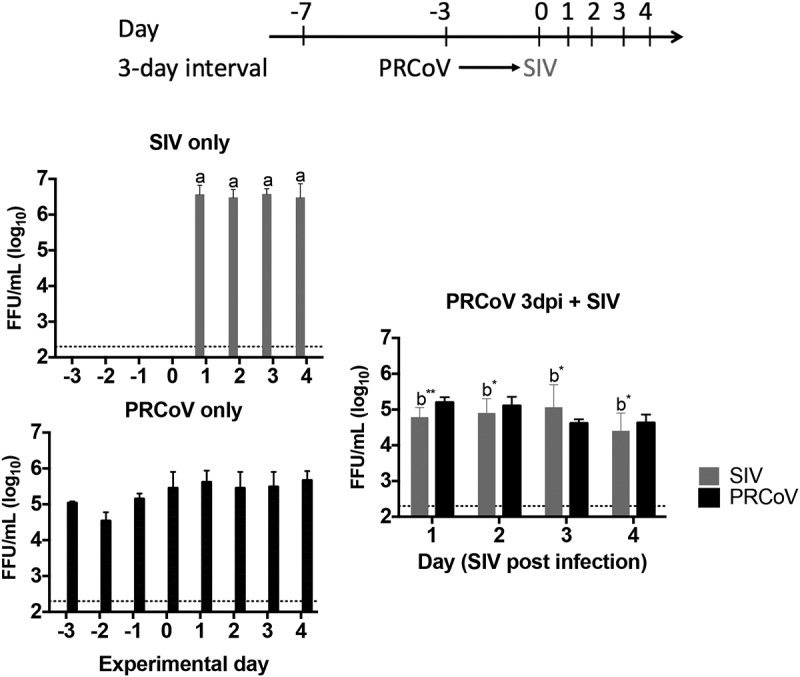
**Virus release from PTECs infected with PRCoV followed by infection with SIV**. PTECs were infected with 1 × 10^3^ FFU PRCoV followed by infection with 4 × 10^4^ FFU of SIV three days later. Control PTECs were infected with SIV or PRCoV only. Viruses released from the apical side were harvested at different time points and titrated by focus-forming assay in ST cells and MDCK cells, respectively. The dashed lines indicate detection limits for the assays. The results are shown as means ± SEM and significance. a, b significant differences between groups are indicated with different letters (* *p* < 0.05 and** *p* < 0.01)

To investigate whether PRCoV infection affects secondary infection by SIV, PTECs were first infected with 1 × 10^3^ FFU PRCoV and then infected with 4 × 10^4^ FFU SIV 3-days later (Figure 5). As shown in Figure 5 and Table 1, the inhibition of SIV growth was detected from day 1 post-infection (*p* < 0.01) and a slight inhibition on 2 dpi to 4 dpi, compared with mono-infection filters (*p* < 0.1). The inhibition of SIV by PRCoV is much less pronounced at the 3-day-interval when compared with the inhibition of PRCoV by SIV (Figure 1 and Table 1).

## Discussion

This study aimed to apply an *invitro* cell culture system of differentiated porcine respiratory epithelial cells to gain insights into the viral interference between infections by coronavirus and influenza virus. This combination of viruses has been analyzed previously with differentiated respiratory epithelial cells only using porcine precision-cut lung slices as culture model [9]. We applied a porcine ALI culture system that has been used so far only to analyze viral-bacterial co-infection [11], but not to investigate viral-viral coinfections. The advantage of the ALI culture system that it has a regeneration capacity that is characteristic for the airway epithelium and enables the analysis of infections that are three or seven days apart.

The ability of the respiratory epithelial cells to regenerate is very important when analyzing influenza virus infections because these viruses have a strong effect which is evident in a dramatic loss of ciliated cells [16]. However, the loss will be compensated by other cells, e.g. basal cells that start to differentiate into specialized cells [16]. Though it takes more than a week until the cells are well–differentiated and have developed cilia, the cell layer remains intact and maintains its barrier function as evident from the transepithelial electrical resistance (TEER) which is unaffected by the virus infection [16]. Results obtained from infection of ALI cultures by influenza viruses have relevance also for understanding natural infections. Pathogenicity was found to be correlated with the loss of ciliated cells in infection of ALI cultures [25–27], i.e. the more pathogenic viruses showed an enhanced virulence in ALI cultures. This effect of SIV was also observed in our study. In contrast to SIV, we did not observe a detrimental effect of PRCoV infection on ALI cultures [21]. This difference may be explained – at least in part– by the different cell tropism of the two viruses. Influenza viruses are very efficient in infecting ciliated cells which are the majority of cells among a differentiated airway epithelium [16]. On the other hand, PRCoV preferentially infects non-ciliated cells and among them the non-mucus-producing cells [21].

Areas in an airway epithelium that are infected by influenza viruses are characterized by the loss of ciliated cells due to apoptosis [16]. The loss of cells is compensated by basal cells that initiate a differentiation process [16]. As mentioned above, these cells can contribute to the maintenance of the barrier function. However, in the transition time, until ciliated cells are generated, they cannot contribute to the mucociliary clearance function. As a consequence, secondary bacterial infections may be facilitated [16,20]. The lack of cilia not only prevents the physical removal of virus particles from the cell surface, it may also facilitate the access of other microorganisms to the cell surface by exposing cell surface components that mediate binding of viruses or adhesion of bacteria [20]. Another feature of the cells in the transition phase is that they have different surface properties. This difference has been revealed by lectin staining. While ciliated cells mainly express α2,6-linked sialic acid on the cell surface, this sugar is predominantly present in the α2,3-linkage type on regenerating cells [16]. The difference also applies to the expression of pAPN, the cellular receptor of PRCoV. Compared to well-differentiated airway epithelial cells, expression of porcine APN is enhanced in areas of infected cells which according to our previous studies are regenerating cells [21]. A similar finding was also noted concerning the expression of ACE2, the cellular receptor for SARS-CoV [28].

Because of the difference in the expression of pAPN, one might expect that regenerating cells are more susceptible to infection by PRCoV. However, prior infection by SIV which resulted in the generation of a substantial number of regenerating cells, did not enhance secondary PRCoV infection. On the contrary, it completely inhibited the coronavirus infection when the virus was applied at the same day or three days after the primary infection. As treatment of ALI cultures with poly-IC also resulted in an inhibition of the infection by PRCoV, we assume that the SIV-induced inhibition of PRCoV infection is due to a similar mechanism, i.e. it may be related to the innate immune response induced by the SIV infection. Our assumption is supported by the finding that PRCoV replication is inhibited in ST cells that have been transfected with SIV RNA, a known inducer of IFN (Fig. S3). When primary and secondary infection in ALI cultures were separated by a time interval of seven days, infection by PRCoV was only partially inhibited. The low level of coronavirus infection under these conditions may in part be explained by the gradual disappearance of the innate immune response [29,30]. Another factor supporting this infection may be the increased surface expression of the cellular receptor for PRCoV, aminopeptidase N after the longer time for cell regeneration. In the future it will be interesting to analyze bacterial infections that result in the loss of ciliated cells and in the induction of a regeneration process. As the innate immune response induced by bacterial infection is not expected to have a protective effect against viral infections, secondary infection may not be inhibited but rather be enhanced because of the increased surface expression of pAPN on the surface of the regenerating cells.

Time-dependent interference in viral-viral co-infections involving influenza viruses have relevance also in natural infections. At the epidemiological level, a seasonal peak incidence of influenza virus infection may delay the expected peak incidence to HCoV–NL63 and other respiratory viruses infection [31–33]. It will be interesting to find out whether the current seasonal influenza viruses interfere with this coronavirus and delay or prevent infection [34,35].

The different order of co- and super-infections caused different outcomes [36]. Compared to a pronounced inhibitory effect after prior infection of SIV, primary infection of ALI cultures by PRCoV caused only a partial inhibition of the SIV infection at three-day interval. The lower efficiency of PRCoV in preventing secondary virus infection may be explained by the different cell tropism of SIV and PRCoV. As the latter virus has a preference for non-ciliated non-mucus-producing cells, it infects a lower number of cells and as a consequence induces a weaker innate immune response. Another possible explanation is that SIV induces a more robust cytokine and chemokine response in pigs than does PRCoV [29,30].

Our study provides new insights how SIV and PRCoV interact in a co- and super-infection scenario on the cellular and molecular level. Such data cannot be obtained with immortalized cells and they are necessary to understand the pathogenicity in co-infected animals. So far, the experimental data about co- and super-infection of pigs by SIV and PRCoV are very limited. In a study where both viruses were concurrently applied to pigs, either virus was isolated less frequently from co-infected animals as compared to mono-infected ones, but no difference was detected in the pathogenicity of mono- and co-infected animals [7]. Another study analyzed the effect of a primary PRCoV infection on a secondary SIV infection at a two- or three-day interval [8]. Here, virus was isolated from nasal swabs after single infections was more frequently as compared to isolation from dual-infections. In both studies, the level of detectable infectious virus in co-infected pigs was found to be reduced which is consistent with our results obtained with ALI cultures and thus can be explained by the host innate immune response. These findings indicate that results obtained with ALI cultures have relevance for animal infections. The different results of the two studies concerning the pathogenicity may be related to the different experimental protocol. For a better understanding more experiments are required. For this purpose, studies with ALI cultures may also be helpful. In our analysis presented here, we focused on the airway epithelium. A limitation of the ALI culture model is that it does not contain immune cells. Therefore, the inflammatory response induced by the immune cells cannot be determined. In the future, it will be interesting to include immune cells such as macrophages or dendritic cells. Such studies will be possible after successful establishment of a co-culture system comprising both epithelial and immune cells.

## Supplementary Material

Supplemental Material
