# Supplementary material for: Time-dependent viral interference between influenza virus and coronavirus in the infection of differentiated porcine airway epithelial cells
Source: Virulence. 2021 May 25;12(1):1111–21. doi: 10.1080/21505594.2021.1911148 (PMC8162253; doi:10.1080/21505594.2021.1911148)
Supplement: Supplemental Material [file KVIR_A_1911148_SM9846.docx]

**Fig. S1.**


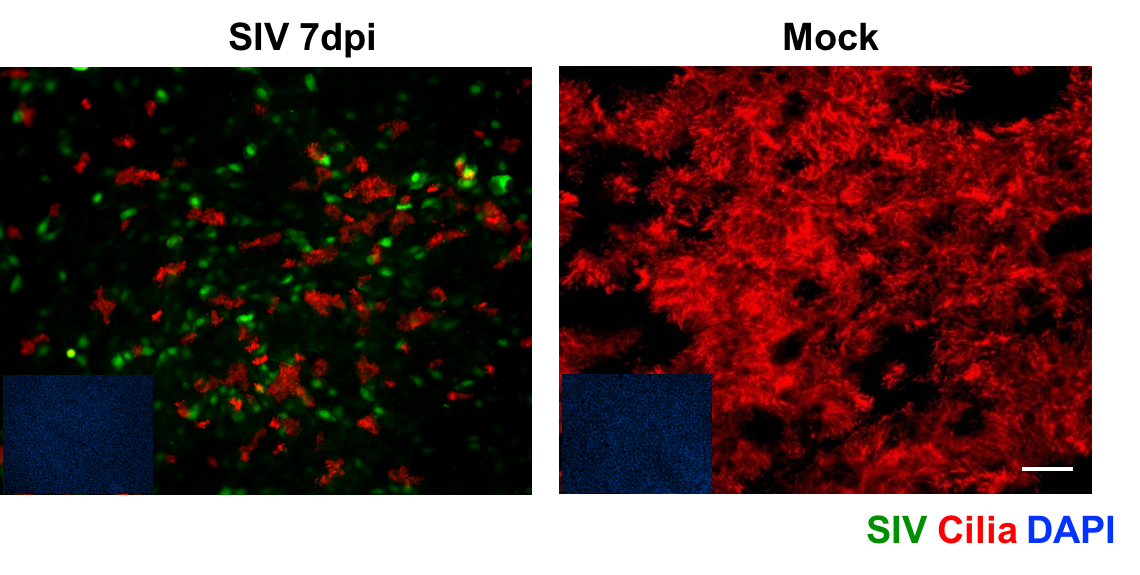


Fig. S1. Immunofluorescent staining of porcine well-differentiated airway epithelial cells at 7 days post infection by SIV. PTECs were inoculated with SIV from the apical side at an 4X10^4^ FFU and fixed at 7 dpi. PTEC cultures were stained for viral nucleoprotein (green), cilia (red), and nuclei (blue).

**Fig. S2.**

Fig. S2. Virus release from porcine primary tracheal epithelial cells (PTECs) infected by swine influenza virus, followed at intervals of 0 or 7 days by secondary infection with porcine respiratory coronavirus (PRCoV). For primary infection, a dose of 4X10^4^ FFU SIV was applied; for secondary infection, PRCoV was applied at 1X10^3^ FFU PRCoV 0 or 7 days later. Viruses released from the apical side were harvested at different time points and titrated by focus-forming assay in ST cell. The dashed lines indicate the detection limits of the assays. The results are shown as means ± SEM.

**Fig. S3.**


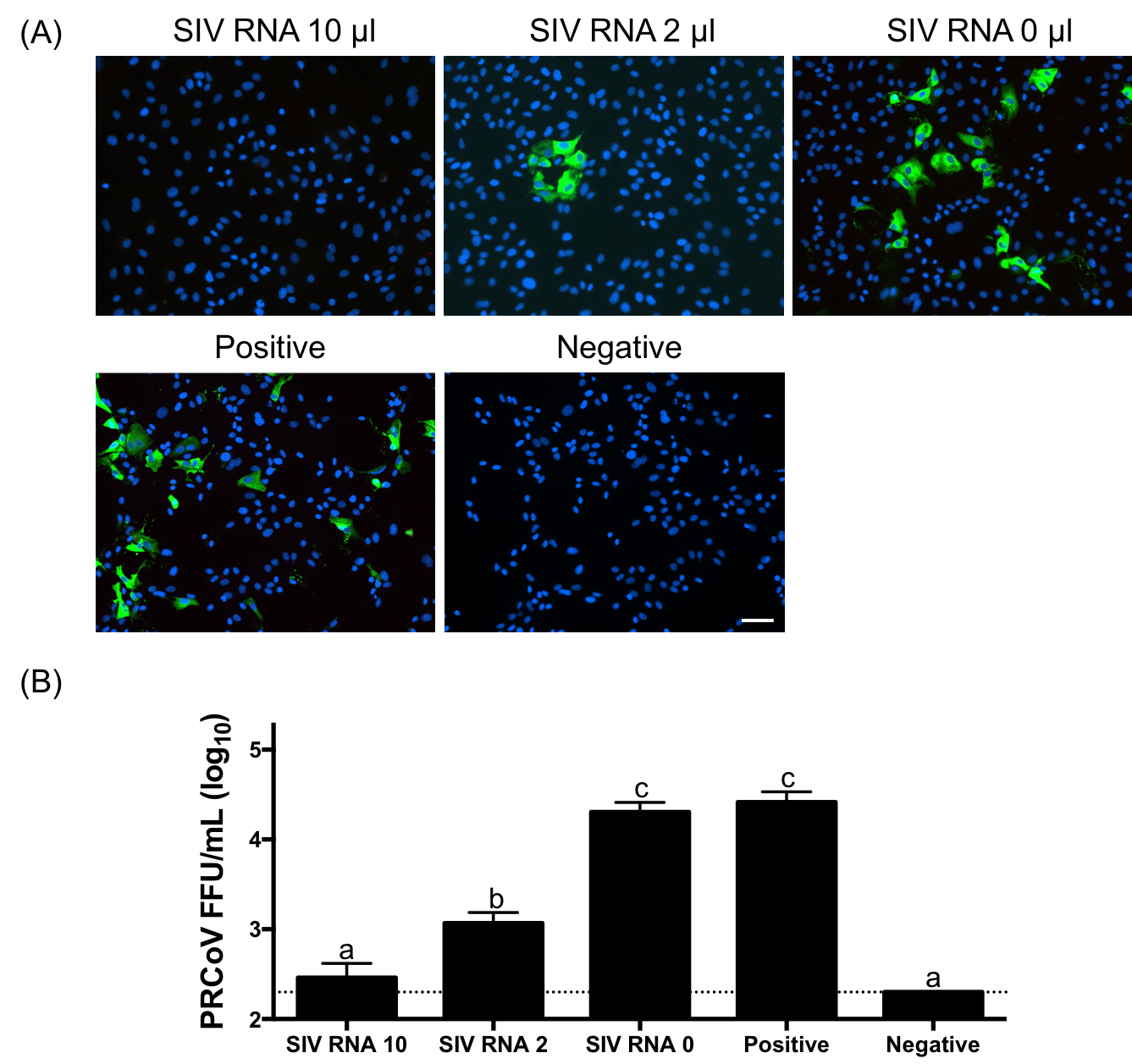


Fig. S3. Replication of PRCoV in swine testicular (ST) cells after prior transfection of SIV RNA. ST cells were transfected with 10 μl, 2 μl, or 0 μl (lipofectamine 2000 only) SIV RNA followed by infection with 0.03 MOI PRCoV. “Positive” indicates the positive control of virus infection indicating that ST cells were infected by PRCoV in the absence of lipofectamine 2000. “Negative” indicates the uninfected control cells. (A) Immunofluorescent staining of pre-transfected ST cells at 2 days after PRCoV infection. Green: viral nucleoprotein. Blue: nuclei (B) Supernatants were harvested at 1 day after PRCoV infection and titrated for the presence of virus by focus-forming assay in ST cells. The dashed lines indicates the detection limit of the assays. The results are shown as means ± SEM. a, b, c significant differences between groups are indicated with different letters.
